# Decision-Making Support for People With Alzheimer’s Disease: A Narrative Review

**DOI:** 10.3389/fpsyg.2021.750803

**Published:** 2021-11-12

**Authors:** Weiyi Sun, Teruyuki Matsuoka, Jin Narumoto

**Affiliations:** Department of Psychiatry, Graduate School of Medical Science, Kyoto Prefectural University of Medicine, Kyoto, Japan

**Keywords:** Alzheimer’s disease, decision-making, cognitive dysfunction, emotional change, feedback, explicit advice

## Abstract

The proportion of people with dementia has been increasing yearly, and the decision-making capacity of these people has become a major concern in fields such as the financial industry and in medical settings. In this narrative review, we discuss decision-making in people with Alzheimer’s disease (AD), and we propose the support for decision-making in people with AD, especially financial and medical decision-making. We summarize several hypotheses and theories on the decision-making capacity of people with AD. These include the frontal lobe hypothesis, physiological theory, dysfunction of the hypothalamic-pituitary-adrenal (HPA) axis, and the Person-Task-Fit (PTF) framework. Both internal and external factors can affect decision-making by people with AD. Internal factors are affected by changes in the brain and neurotransmitters, as well as alterations in cognitive ability and emotion. External factors include task characters, task contents, and situation influence. Since feedback has a significant effect on decision-making capacity, a series of suggestions may be helpful to improve this capacity, such as explicit advice, simple options, pleasant rewards, the Talking Mats approach, memory and organizational aid, support by caregivers, cognitive training and feedback. Thus, in providing decision-making support for people with AD, it is important to identify the internal and external factors that impair this process and to deal with these factors.

## Introduction

About 50 million people worldwide suffer from dementia, and nearly 10 million new cases were occurred every year. In Japan, the number of people with dementia is more than 4.6 million, and dementia has become a major concern among older people (World Health Organization, 2020). Since elderly people in Japan comprise a large percentage of the population, the problems caused by dementia, especially Alzheimer’s disease (AD) which is the most common type of dementia, have a profound impact on the whole society.

The body of decision-making theory is that someone prefers A to B (or vice versa) when someone may be placed into different two states, A and B (Edwards, 1954). In decision-making in game tasks, the responses of people with AD are less consistent than those of healthy elderly people (de Siqueira et al., 2017). The rate of select the disadvantage decisions under ambiguity risk was significantly higher in mild cognitive impairment (MCI) and AD than in controls, and this disadvantage decisions was linked to the reduced ability to perform a variety of task about activities of daily living, cognition, interpersonal relationship, and emotion, and the poor awareness of limitations (Jacus et al., 2018). People with AD made more risky decisions than controls when they walked across the road (Fang et al., 2018). Therefore, people with AD face difficulties with decision-making in various situations.

Financial capacity includes a broad spectrum of conceptual, pragmatic, and judgment abilities (Marson et al., 2000) and has four associated factors: basic monetary knowledge and calculation skills, financial judgment, conceptual knowledge, and procedural knowledge (Gerstenecker et al., 2018). Decreased financial decision-making ability is linked to vulnerability of financial exploitation in older people (Lichtenberg et al., 2016). Medical decision-making capacity comprises four aspects of understanding, appreciation, reasoning, and expression of choice (van Duinkerken et al., 2018). Financial capacity is mildly impaired even in normal aging (Bangma et al., 2017) and MCI (Marson et al., 2009), and this capacity gradually declines as AD progresses (Marson et al., 2009), which is consistent with a recent systematic review (Bangma et al., 2021). In the financial decision-making task, the proportion of risky choices in people with AD was significantly higher than in controls, especially for opportunities to gain money rewards (Ha et al., 2012). People with AD also have lower medical decision-making capacity compared to subjects with normal cognitive function or MCI (Palmer et al., 2017; Carabellese et al., 2018; van Duinkerken et al., 2018). Some reviews suggest a difference between financial capacity and medical decision-making capacity because the former is a multidimensional activity, whereas the latter is mainly a verbally mediated activity (Moye and Marson, 2007; Widera et al., 2011; Marson, 2013; Bangma et al., 2021). A systematic review of financial decision-making showed involvement of global cognition, processing speed, numeracy, verbal memory, and working memory in people with AD (Bangma et al., 2021), while a similar review found that global cognition, episodic and working memory, executive functions, and linguistic abilities are linked to medical decision-making in people with AD (van Duinkerken et al., 2018). Since the cognitive functions related to financial and medical decision-making partially overlap, there might be a common support method for financial and medical decision-making in people with AD. In an aging world, finding ways to support the decision-making and improve well-being of persons with AD is becoming increasingly important. Financial and medical decision-making support is one of the challenges in a clinical setting. In this narrative review, we discuss decision-making in people with AD, and we propose support for decision-making, especially in the financial and medical areas, for people with AD.

## Theories of Decision-Making in Ad

There are several theories and hypotheses explaining the decision-making mechanism of AD. In the frontal aging hypothesis, the age-related changes of brain are more applicable to age-related decision-making changes. This theory suggests that frontal brain regions experience greater deterioration than other regions as a result of aging, causing a significant reduction in frontal lobe function (West, 1996). Brain regions associated with the frontal lobe, including the ventromedial prefrontal cortex (Denburg et al., 2007; Kennerley and Walton, 2011; Samanez-Larkin and Knutson, 2015), orbitofrontal cortex (Camerer, 2007), ventral striatum (Camerer, 2007), paracingulate (Camerer, 2007), dorsolateral prefrontal cortex (Camerer, 2007), nucleus accumbens (Samanez-Larkin and Knutson, 2015), and anterior insula (Samanez-Larkin and Knutson, 2015), are involved in decision-making. Frontal, temporal, and parietal regions also play important roles in decision-making and are especially vulnerable to age-related change in volumetric analyses of gray matter (Good et al., 2001; Driscoll et al., 2009; Kennedy et al., 2009; van Duinkerken et al., 2018). AD is characterized by loss of neurons and synapses in the cerebral cortex and in certain subcortical areas. The main sites involved are the hippocampus, temporal lobe, parietal lobe, part of the frontal lobe, and cingulate gyrus (Wenk, 2003). Frontal lobe dysfunction appears with progression of AD, but frontal dysfunction is prominent even at the early stage in frontal variant AD (Sawyer et al., 2017). The prevalence of frontal variant AD is 2–3% of all AD cases (Sawyer et al., 2017). However, since 10–40% of people with clinically diagnosed behavioral variant frontotemporal dementia (bvFTD) have an AD pathology (Ossenkoppele et al., 2015), frontal variant AD is often misdiagnosed as bvFTD. In patients with AD, atrophy of the temporal and parietal lobes was associated with poorer performance in gambling task (Kloeters et al., 2013), and atrophy of the medial prefrontal cortex and the corresponding decline in attention were related to decreased financial capacity (Stoeckel et al., 2013). Therefore, decision-making may be affected by neuropathological changes that occur in people with AD.

In physiological theory, altered decision-making is linked to neurotransmitters such as dopamine (Mohr et al., 2010; Eppinger et al., 2011; Samanez-Larkin and Knutson, 2015), serotonin (Mohr et al., 2010; Eppinger et al., 2011), norepinephrine (Mohr et al., 2010; Samanez-Larkin and Knutson, 2015), and glutamate (Samanez-Larkin and Knutson, 2015). Samanez-Larkin and Knutson (2015) proposed an affect-integration-motivation framework to explain the involvement of affective and motivational circuits in decision-making. Dopaminergic, noradrenergic, and glutamatergic neurons are involved in this circuits. Patients with AD have lower dopamine and dopamine receptor levels, and changes of neurosecretion of the dopamine system are related to progression of AD (Pan et al., 2019). Similarly, the raphe nucleus, which is the main serotonin-producing area of the brain, is significantly affected by AD pathology (Chen et al., 2000). Loss of noradrenergic neurons greatly exacerbates AD pathology and leads to progression of the stage of AD (Gannon et al., 2015). AD pathology causes progressive degeneration of locus coeruleus noradrenergic neurons, which is linked to impairment of global cognition, episodic memory, working memory, and visuospatial ability (James et al., 2020). Glutamate and its receptors are also involved in synaptic plasticity and the etiology of neurodegenerative diseases such as AD (Wang and Reddy, 2017). These physiological alterations in AD are likely to lead to changes in the decision-making process.

Dysfunction of the hypothalamic-pituitary-adrenal (HPA) axis caused by stress response might also be a cause of AD (Brureau et al., 2013; Park et al., 2015). Abnormal secretion by the HPA axis increases levels of glucocorticoid, which have harmful effects on the hippocampus (Pineau et al., 2016). As a metabolite of dopamine, hydroxytyrosol can ameliorate the negative effects of impulsivity, which has been shown as a suboptimal form of cost-benefit decision-making in an AD mouse model (ArunSundar et al., 2018).

The Person-Task-Fit (PTF) framework proposes that individual decision-making capacity is influenced by personal characteristics (e.g., age, education, crystallized and fluid intelligence, cognitive function, and affective skills), as well as characteristics of task (e.g., familiarity, information complexity) and context (e.g., sociocultural values and stereotypes; Finucane and Lees, 2005).

There are two types of risk situations in decision-making: ambiguity risk and objective risk. In ambiguity risk, probability cannot be calculated and the expected effects of different options cannot be estimated because explicit information is not presented (Sinz et al., 2008; Schiebener and Brand, 2015). Decisions made under ambiguity risk seem to be more related to the ventromedial and orbital prefrontal cortex than the dorsolateral prefrontal cortex (Brand et al., 2006). A decision situation under ambiguity risk activates nondeclarative dispositional knowledge associated with previous emotional experience of similar situations, which assists the reasoning process, representation of future outcomes, and the decision (Brand et al., 2006).

In objective risk, explicit information is presented, and the results of choices can be predicted by well-defined or estimable probabilities (Sinz et al., 2008; Schiebener and Brand, 2015). The previous study proposed a model, in which decision-making under objective risk conditions is mediated by an impulsive system and a reflective system (Schiebener and Brand, 2015). The impulsive system is involved in emotional reactions, conditioning, and somatic activity, while the reflective system includes executive functions, working memory, and reasoning. When a decision is made, the two systems are activated at the same time, but only one is triggered as the leading processing mode. Which system is dominant depends on individual predispositions and the situational condition. After the decision is made, feedback is processed through both systems, and this influences which system dominates the next decision process. Thus, both internal factors such as impulsive and reflective systems and external factors such as information about the decision situation, situational induced states, and external influences can affect decision-making under objective risks (Schiebener and Brand, 2015).

## Factors Associated with Decision-Making in Ad

Based on the theories of decision-making in AD, the internal factors, including cognitive impairment and emotional change caused by biological change, and external factors might be involved in the impairment of decision-making in AD.

## Cognitive Impairment

People tend to make decisions based on what they have learned in the past, and cognitive ability is especially important. Older people tend to use simpler strategies that need lower cognition and take a longer time to complete when thinking about things (Mata et al., 2007). In other words, older people tend to use less information when making a decision, but this leads only to a small loss in decision quality (Mata and Nunes, 2010).

Fluid intelligence refers to perception and processing of problems, while crystallized intelligence refers to use of experience and knowledge learned in the past (Cattell, 1987). Fluid cognitive ability is linearly and negatively correlated with age, whereas crystallized ability increases non-linearly and tends to be stable in late middle age (Agarwal et al., 2009). Therefore, fluid intelligence of elderly people is lower than that of younger adults, while crystallized intelligence is higher. People with mild AD may have a more pronounced decline in crystallized intelligence than people without AD, but no significant difference in fluid intelligence (Matsuda and Saito, 1998). However, some studies have found that fluid intelligence declines in people with AD, and that the discrepancy between impairment of fluid and crystallized intelligence increases with progression of AD (McCarthy et al., 2005; Dierckx et al., 2008). This discrepancy may be correlated to cortical thinning and Aβ deposition (McDonough et al., 2016). Moreover, even people with preclinical AD, as defined by amyloid positron emission tomography, had a greater decline in fluid intelligence, but not in crystallized intelligence, compared to cognitively normal older adults (Harrington et al., 2018).

Generally, better Mini-Mental State Examination scores and memory, frontal lobe, and language functions are correlated with better medical decision-making capacity (van Duinkerken et al., 2018). Various functions are involved in medical decision-making, including understanding information, developing rationales for a decision, appreciating the consequences of the choice, making a reasonable decision, and expressing a choice (van Duinkerken et al., 2018). Compared to healthy older adults, people with dementia have impairment in the understanding and reasoning processes (Moye et al., 2006). Such declines in cognitive impairment and intelligence affect decision-making and can lead to people with AD to need to make simpler and more straightforward choices compared to healthy elderly people.

Memory of people with AD is important to their decision-making capacity. Bayard et al. (2015) confirmed the role of declarative memory in decision-making of patients with AD under ambiguity risk, using experiments that indicated that patients with AD and MCI tended to make disadvantageous decisions compared to a control group because they could not fully understand explicit knowledge. This is consistent with another study demonstrating that decision-making problems in AD patients under ambiguity risk might be due to memory impairment (Alameda-Bailen et al., 2017). A third study also found that poor performance of decision-making under risk is closely correlated with memory impairment, as well as with lower executive function (Sun et al., 2020). In financial decision-making, semantic memory is linked to financial knowledge, while episode memory and visuospatial ability are related to numeracy (Gamble et al., 2015). Working memory (Stormoen et al., 2014; Thalen et al., 2017), short-term verbal memory (Earnst et al., 2000; Thalen et al., 2017), verbal knowledge (Stormoen et al., 2014), and semantic knowledge (Earnst et al., 2000) might be involved in medical decision-making capacity. Thus, poor memory may affect decision-making in patients with AD.

The process of decision-making is required to coordinate several simultaneous cognitive and behavioral processes (Gleichgerrcht et al., 2010). Executive function is involved in decision-making performance under ambiguity risk and objective risk in patients with AD (Sinz et al., 2008), and a separate study linked executive dysfunction to poor decision-making under risk (Delazer et al., 2007; Sun et al., 2020). Executive function is also associated with medical decision-making capacity in AD (Okonkwo et al., 2008; Lui et al., 2010).

Delay discounting (DD) can be defined as the depreciation of value of a reward related to the time that it takes to be released. This phenomenon can be evaluated as the impulsiveness of subjects choosing between rewards available only after some length of time and smaller rewards that are available immediately (Matta et al., 2012). DD in people with MCI and mild AD has been related to lower verbal intelligence, but not to age, general cognitive ability, functional ability, living status, or marital status (Thoma et al., 2017). People with MCI may display significantly higher levels of DD, which indicates that they tend to respond to smaller rewards impulsively (Lindbergh et al., 2014). Compared to MCI patients and older adults, AD patients display even higher DD (Lebreton et al., 2013; Thoma et al., 2017; El Haj et al., 2020; Geng et al., 2020). These findings are probably related to impaired autobiographical memory in patients with AD (El Haj et al., 2020) and executive function deficits in patients with MCI (Geng et al., 2020). Patients with AD may be more prone to immediate rewards due to a shrinking hippocampus (Lebreton et al., 2013) and amygdala (Manuel et al., 2020). However, Bertoux et al. (2015) found opposite results of no difference in impulsivity between AD patients and controls. Recently, Beagle et al. (2020) demonstrated that patients with AD were less influenced by information offered during the choice than controls, although impulsivity in patients with AD was not different from controls. The different types of reward may affect the response, since AD patients have been shown to have faster reaction times for monetary loss than monetary gain, and for social wins compared to social losses, which is opposite to findings in bvFTD (Perry et al., 2015). The condition might be also associated with the response because patients with AD were significantly more impulsive than controls only in the situations that caused negative emotions (Manuel et al., 2020). In general, executive function and DD reflects the ability of AD patients to make decisions.

## Emotion and Neuropsychiatric Symptoms

The affect-integration-motivation framework, as mentioned above, anticipates gain and loss, integrates value, and facilitates the next action (Samanez-Larkin and Knutson, 2015). Older adults have stable affective experience as they age (Carstensen et al., 2011) and elderly people tend to deal with negative emotions in decision-making better than younger adults (You et al., 2019), although they show lower emotional and neurological sensitivity to anticipated economic losses (Samanez-Larkin and Knutson, 2015).

In patients with AD, behavioral and psychological symptoms of dementia (BPSD) and cognitive impairment influence decision-making. Apathy and affective and emotional symptoms are common in AD (Alzheimer’s Association, 2016), and patients with AD have more severe impairments in emotion perception, compared to healthy older adults and patients with MCI (Elferink et al., 2015). Bayard et al. (2014) showed that a severe apathy, especially impairment of action initiation, appeared to be associated with disadvantageous decision-making under ambiguity risk in AD and amnestic MCI. Fong et al. (2017) found that patients with AD, as well as healthy controls, had increased emotional arousal and hesitated to cause harm to other people in moral decision-making, while patients with bvFTD had decreased emotional arousal and responded faster to moral dilemmas. A recent review of everyday decision-making in dementia indicated the importance of emotion regulation, although, the association with depressive symptoms was inconclusive (Perach et al., 2020). Recently, depressive symptoms in patients with AD increased the rejections of unfair offers in decision making task (Yerstein et al., 2020). In medical decision-making, AD patients with apathy and delusions have impaired ability in expression of choice, whereas patients with depression had better performance in this regard (Bertrand et al., 2017), and AD patients with euphoria have a low reasoning performance (Bertrand et al., 2017). Anxiety in patients with AD affected the decision-making capacity in a real informed consent situation (Kato et al., 2021). Thus, the influence of emotion and neuropsychiatric symptoms on decision-making in people with AD is also crucial.

## External Factors in Decision-Making

In evaluation of decision-making capacity, the influence of external factors such as task characters and contents cannot be ignored. In the PTF framework, task characters include information complexity, familiarity, framing, instrumental nature, and affective engagement; and task contents include sociocultural values, stereotypes, time pressure, and decision support (Finucane and Lees, 2005). For example, Finucane et al. (2005) suggested that increased age and task complexity could lead to greater understanding errors and inconsistencies in decision-making; and Glazer and Karpati (2014) pointed out the influence of cultural values on decision-making. Thus, patients with AD might be able to consent to low-risk content in medical decision-making, but not to high-risk content, which may be more complex and requires higher cognitive function (van Duinkerken et al., 2018). This suggests an influence of information complexity on decision-making in AD.

Patients with AD have difficulty in decision-making under both ambiguity and objective risk situations, since these patients use less negative feedback and select more disadvantageous options under these situations (Delazer et al., 2007; Sinz et al., 2008; Bayard et al., 2015; Jacus et al., 2018; Sun et al., 2020).

## Support For Decision-Making

It is important for people with dementia to make daily decisions because this is associated with their well-being and quality of life (Davis et al., 2017). Support for decision-making differs depending on the stage of AD (Slyer et al., 2018). In the early stage, people with AD prefer to make decisions while collaborating with caregivers; in the mid-stage, caregivers need to be more involved in the decision-making; and in the late stage, caregivers should serve as surrogate decision-makers (Berry, 2014; Davis et al., 2017; Slyer et al., 2018). It should be noted that a caregiver may not always make the best judgment, and this may deprive a patient of the opportunity to make a decision (Davis et al., 2017).

The advice that is most effective for healthy subjects to support decision-making under risk includes expanded instruction, presentation of probabilities, presentation of maximum gain and loss, explicit advice, and additional test trials; and decision support using explicit advice is most supportive to improve the quality of decisions made under objective risk, especially for subjects with poor working memory and executive function (Schiebener et al., 2013). Engelmann et al. (2009) reached a similar conclusion, suggesting that expert advice significantly improves financial decision-making capacity under risk in healthy people. Since responses in most brain areas were significantly greater without advice compared to with advice, the improved decision-making may be due to the experts advice unloading the calculation of the value of decision options from the brain (Engelmann et al., 2009).

Similarly, decision support can improve the decision-making capacity of patients with AD. Simplifying the information might be also useful to support the comprehension of people with AD. A study demonstrated the effectiveness of simplifying the information in comprehension of consent form in people with mild to moderate AD (Moro et al., 2020). However, the readability of vignette did not improve the medical decision-making in people with mild AD (Thalen et al., 2017). Mueller et al. (2019) found that patients with mild AD with explicit information about the risks of a just-made decision in a decision-making task made fewer risky choices and more inclined to make favorable decisions, compared to those without this support. Memory and organizational aid summarizing the key points of information have been shown to improve the consent capacity of patients with very mild to early moderate AD (Rubright et al., 2010). In assessment using the MacArthur Competence Assessment Tool for Clinical Research (MacCAT-CR), understanding was significantly and appreciation was slightly improved by memory and organizational aid, while reasoning and expression of choice were unchanged (Rubright et al., 2010). Therefore, support using explicit advice and memory and organizational aid is an excellent method to improve the decision-making capacity of people with AD.

Feedback is also very important for an individual to make a decision. Encouragement of reasoning about given choices and possibilities of a decision situation can improve decision-making under explicit risk conditions (Pertl et al., 2017). However, corrective feedback using video showing important information did not improve the medical decision-making in mild to moderate AD (Palmer et al., 2018). Cognitive training might also improve decision-making, since executive-function and numerical training are effective for decision-making in patients with MCI under risk conditions (Burgio et al., 2018). In addition, enhanced information exchange between people with dementia and their caregivers can also improve decision-making (Murphy and Oliver, 2013). Murphy and Oliver (2013) designed a communication framework called Talking Mats that helps individuals organize their thoughts and enhance information exchange by providing visual cues. People with dementia and their caregivers who used Talking Mats were found to feel more connected to each other and to be happier because each felt that his or her ideas were being heard and taken into account (Murphy and Oliver, 2013).

As cognitive function declines, patients with AD become unable to make decisions independently. In a clinical setting, informed consent of patients with severe AD is a serious problem (Hegde and Ellajosyula, 2016). Legal support plays an important role in maintaining the right of patients to informed consent. Laws on informed consent vary from country to country, but mentally incapacitating diseases such as dementia do not result in loss of legal rights (Guzik-Makaruk et al., 2019). However, in the late stage of AD, a surrogate such as a caregiver needs to anticipate a patient making a decision (Slyer et al., 2018). In terms of surrogate decisions, implementation standards vary from country to country due to different legal structures (Tsoh et al., 2015). Even in Asia, where laws are partially common among countries, the roles of physicians, family members, and non-governmental organizations vary due to different cultural and social backgrounds (Tsoh et al., 2015). Therefore, it is considerable to comprehend the preferences and values of a patient and to make decisions when the patients are able to participate in decision-making (Slyer et al., 2018).

## Discussion

**Figure 1:**
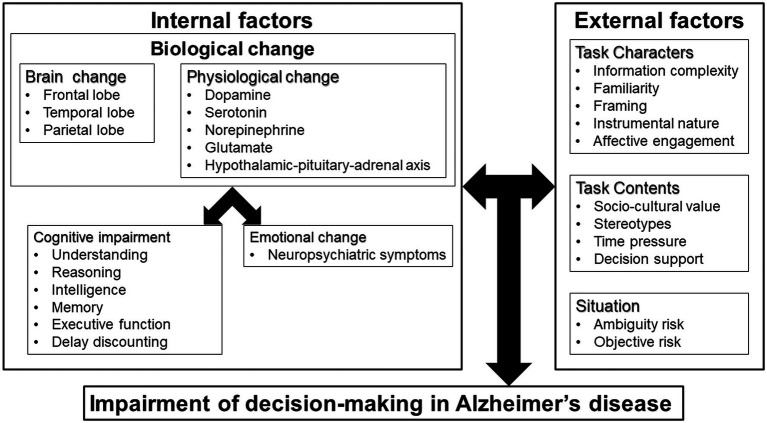
Mechanisms of impairment of decision-making in Alzheimer’s disease (AD).

This narrative review of the theory, influential factors, and support for decision-making by people with AD indicates that impairment of this process occurs as a result of internal and external factors (Figure 1). Internal factors include brain changes, physiological changes, cognitive impairment, and emotional changes; and external factors include information complexity, cultural values, and situation of decision-making.

**Figure 2:**
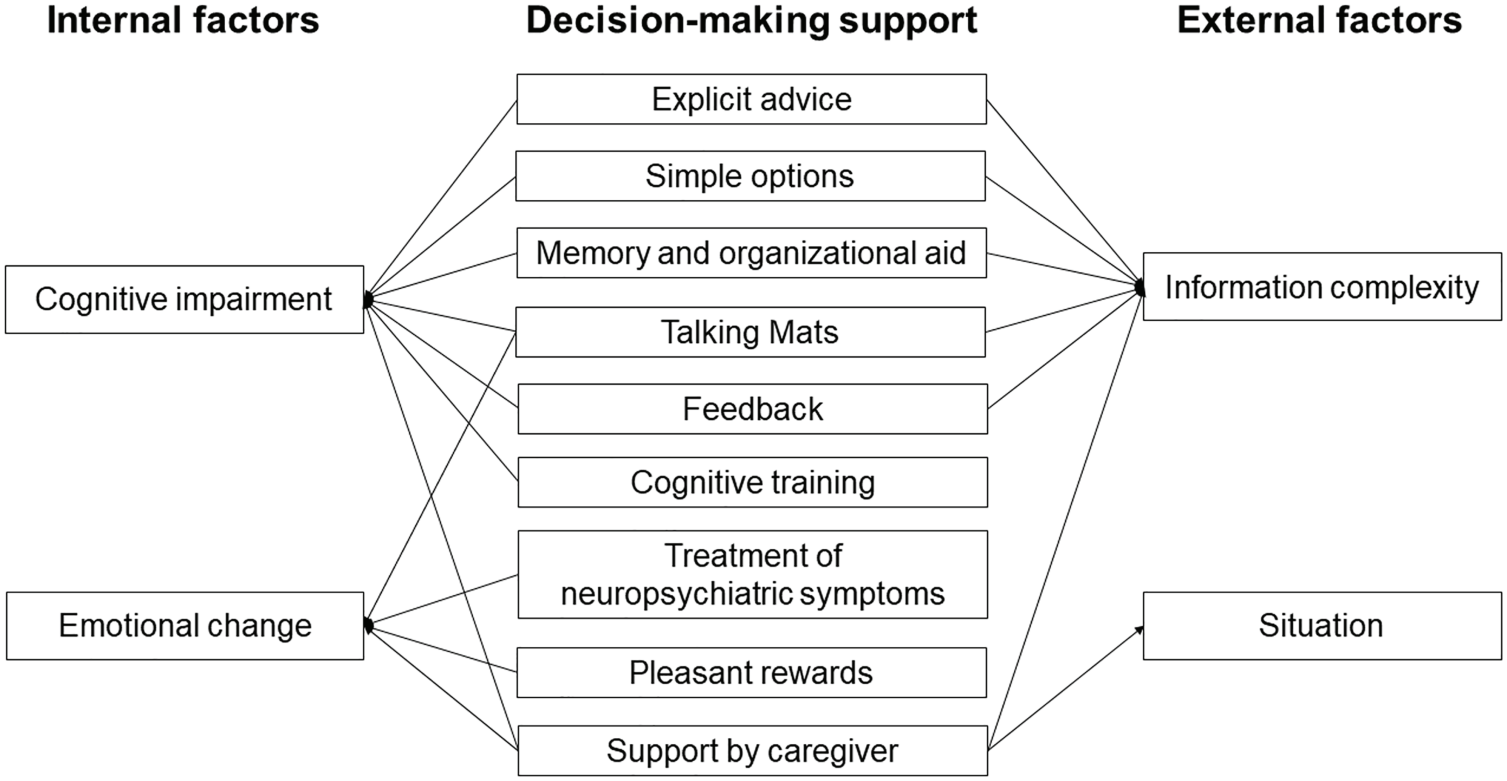
Perspectives on decision-making support for people with Alzheimer’s disease.

Early detection and support for internal factors might be the key to improving the decision-making ability of people with AD (Figure 2). Support for cognitive impairment is important to bring out this ability, including explicit advice and provision of simple options. Support including memory and organizational aids and use of Talking Mats can also compensate for cognitive impairment of people with AD. Since feedback might improve decision-making (Pertl et al., 2017), repeated explanation and feedback may improve the process. Cognitive training might also improve decision-making in the early stage of AD, based on the finding that cognitive training can improve decision-making in patients with MCI (Burgio et al., 2018). Treatment of emotion and neuropsychiatric symptoms might also be useful, since decision-making in people with AD may be affected by their low emotional sensitivity and poor ability to regulate emotion (Samanez-Larkin and Knutson, 2015; You et al., 2019). Therefore, emotional changes through interaction with others using tools such as Talking Mats might improve decision-making. Having a choice of pleasant rewards that are preferred by decision-makers can also be helpful. Support by a caregiver is useful to compensate for cognitive impairment and will reduce anxiety, but this support should be the minimum required to respect the decisions of people with AD.

Interventions for external factors might also be important to support decision-making in people with AD, including support for understanding and reasoning in decision-making processes. Explicit advice, simple options, memory and organizational aid, use of Talking Mats, and feedback are useful to reduce information complexity, and support in ambiguous and risky situations is particularly important for people with AD. Support by a caregiver is also useful to reduce information complexity and anxiety in these situations.

Useful decision-making support might differ in each stage of AD (Table 1). Most support is likely to be useful for people with mild AD. As mentioned above, support by a caregiver should be minimized. As the stage progresses, it becomes difficult for the patient to make a decision by themselves, even if there is support, and substitute decision-making by caregivers becomes important. However, it is also important to support decision-making for people with moderate to severe AD using tools such as memory and organization aids and Talking Mats.

**Table 1:** Type of decision-making support in each stage of Alzheimer’s disease.

Type of support	Stage of Alzheimer’s disease		
	Mild	Moderate	Severe
Explicit advice	✓		
Feedback	✓		
Cognitive training	✓		
Simple options	✓	✓	
Memory and organization aid	✓	✓	
Treatment of neuropsychiatric symptoms	✓	✓	
Pleasant rewards	✓	✓	
Talking mats	✓	✓	✓
Support by caregiver		✓	✓

Since some studies did not show the effectiveness of decision-making support for people with AD (Thalen et al., 2017; Palmer et al., 2018), decision-making support might rather cause their confusion. However, thinking about people with AD and explaining politely when they make decisions are necessary. Thus, in providing decision-making support for people with AD, it is important to identify the internal and external factors that impair decision-making and to deal with these factors.

## Limitation

There is a limitation of application of the results of experimental studies to real life situations. The Iowa Gambling Task is often used to measure ability under ambiguity risk (Buelow and Suhr, 2009). In contrast, the Game of Dice Task is used to evaluate decision-making under objective risk conditions (Brand et al., 2005). Financial decision-making is commonly examined using the financial capacity instrument (FCI), financial assessment and capacity test (FACT), and assessment of capacity for everyday decision-making (ACED; Bangma et al., 2021). The MacArthur Competence Assessment Tool for Treatment (MacCAT-T), MacCAT-CR, and Capacity to Consent to Treatment Instrument (CCTI) are often used for assessment of decision-making in medical and research settings (van Duinkerken et al., 2018). These instruments are commonly used in clinical settings to measure decision-making capacity, but the test scores should be used as a supplement, rather than to supplant a clinical judgment of capacity (Kapp and Mossman, 1996). However, the experimental task may at least partly reflect the real situation. For example, objective risk in experimental studies is similar to the real situation in financial and medical decision-making because the objective risk is presented. Kato et al. (2021) demonstrated that understanding and reasoning were impaired in people with AD in a real informed consent situation, which is consistent with an earlier study using hypothetical vignettes (Moye et al., 2006). Therefore, the results of experimental studies are also useful in considering support for decision-making. On the other hand, experimental studies of ambiguity risk may not be applicable to a real situation. Asset management and stock trading might be similar to the ambiguity risk condition, but these are more complex than typical tasks in ambiguity risk tests.

## Conclusion

In this narrative review, we propose support for decision-making for people with AD. However, the proposed mechanisms of support are largely based on the results of experimental decision-making tasks. Further research is needed to examine the effectiveness of such decision-making support in a clinical setting.

## Author Contributions

WS and TM designed the study, searched and reviewed previous studies, and wrote the paper. JN designed the study and wrote the paper. All authors contributed to the article and approved the submitted version.

## Funding

The study was supported by JST COI (Grant Number: JPMJCE1302).

## Conflict of Interest

The authors declare that the research was conducted in the absence of any commercial or financial relationships that could be construed as potential conflicts of interest.

## Publisher’s Note

All claims expressed in this article are solely those of the authors and do not necessarily represent those of their affiliated organizations, or those of the publisher, the editors and the reviewers. Any product that may be evaluated in this article, or claim that may be made by its manufacturer, is not guaranteed or endorsed by the publisher.
